# Integrated pan-cancer analysis of RNA binding protein HuR investigates its biomarker potential in prognosis, immunotherapy, and drug sensitivity

**DOI:** 10.1371/journal.pcbi.1013374

**Published:** 2025-08-25

**Authors:** Jian Peng, Jichuan Quan, Xiaoru Wang

**Affiliations:** 1 Anhui Institute of Pediatric Research, Anhui Provincial Children’s Hospital, Hefei, Anhui, China; 2 Department of Colorectal Surgery, National Cancer Center/National Clinical Research Center for Cancer/Cancer Hospital, Chinese Academy of Medical Sciences and Peking Union Medical College, Beijing, China; 3 Department of Critical Care Medicine, Second Hospital of Shanxi Medical University, Taiyuan, Shanxi, China; La Jolla Institute for Immunology, UNITED STATES OF AMERICA

## Abstract

**Background:**

While the RNA-binding protein HuR is implicated in individual cancers, its comprehensive diagnostic, prognostic, and immunological roles across diverse cancer types remain unexplored.

**Methods:**

We performed an integrated pan-cancer analysis of HuR using public datasets. This encompassed expression profiling, survival analysis, diagnostic accuracy assessment, immune microenvironment characterization, and drug sensitivity prediction. We investigated HuR’s regulatory mechanisms through pathway correlation and differential gene expression analyses.

**Results:**

HuR expression was consistently elevated across multiple cancers and correlated with poor patient prognosis. It demonstrated high diagnostic accuracy (>85%) via TMB/PD-L1 biomarkers. High HuR expression was associated with an immunosuppressive tumor microenvironment and reduced efficacy of immune checkpoint inhibitors, establishing it as a key immunoregulatory biomarker. HuR also predicted sensitivity to cell cycle inhibitors and other pathway-targeted drugs. Mechanistically, HuR drives malignancy by dysregulating core processes: cell cycle progression, immune evasion, and cellular metabolism.

**Conclusions:**

Our pan-cancer analysis establishes HuR as a consistently upregulated oncogenic driver across malignancies, functioning as a potential universal biomarker for prognosis and diagnosis. Its critical roles in modulating the immune response and predicting therapeutic sensitivity highlight its importance for personalized cancer treatment strategies. HuR orchestrates tumorigenesis and malignant progression by integrally regulating vital cellular processes.

## Author summary

HuR’s pan-cancer role was unclear, hindering targeted therapy development. To address this, we conducted the first integrated analysis of HuR across 33 cancers using data from >10,000 patients (TCGA/CCLE/GTEx). We found HuR is consistently elevated in tumors and predicts poor survival pan-cancer. It achieves >85% accuracy in cancer detection using TMB/PD-L1 biomarkers. High HuR levels create “immune-cold” tumors, reducing immunotherapy effectiveness, and control sensitivity to cell cycle drugs. Mechanistically, HuR acts as a master regulator, dysregulating cell division, immune evasion, and cancer metabolism. This study establishes HuR as a universal biomarker for cancer prognosis and diagnosis, a predictor of drug response, and a multifunctional driver of malignancy. This pan-cancer blueprint now enables prioritization and development of HuR-targeted therapies across diverse cancers.

## Introduction

Cancer remains a formidable global challenge, impacting society, public health, and economies. In 2022, ~ 20 million new cancer cases and 9.7 million deaths were reported worldwide [1,2]. Projections indicate this will rise to 35 million annual new cases by 2050 [1,3]. China mirrors this burden, with 4.82 million new cases and 2.57 million deaths in 2022 [4,5]. The five most prevalent cancers—lung, colorectal, thyroid, liver, and gastric—comprised 57.42% of new diagnoses, while lung, liver, gastric, colorectal, and esophageal cancers accounted for 67.50% of cancer deaths [4]. These statistics highlight cancer’s persistent severity and underscore the urgent need to elucidate molecular mechanisms of tumorigenesis and progression.

RNA-binding proteins (RBPs) comprise a diverse family of proteins that intimately engage with RNA molecules across the entire spectrum of its regulatory metabolic pathways [6]. Their interactions are integral to the intricate network of post-transcriptional processes that govern RNA fate and function [7]. By interacting with RNA, RBPs regulate a wide range of cellular functions, including RNA maturation, transport, localization, and translation, among others [8,9]. Based on their diverse functions, RBPs can be categorized into distinct groups, including Hu-antigen R (HuR), epithelial splicing regulatory proteins, cytoplasmic polyadenylation element binding proteins, heterogeneous nuclear ribonucleoprotein family members, insulin-like growth factor 2 mRNA-binding proteins, the Zfh family of transcription factors, KH-type splicing regulatory protein, La ribonucleoprotein domain family members, Lin-28 homologs, Musashi protein family, Pumilio protein family, Quaking, RNA-binding motif proteins, Src-associated substrate in mitosis of 68 kDa, serine and arginine-rich splicing factors, T-cell intracellular antigens, and Upstream of N-Ras [10,11]. In the context of cancer, aberrant expression of RBPs were commonly observed, underscoring their substantial potential as biomarkers for prognosis and diagnosis. Furthermore, the effect of RBPs on tumor progression relied heavily on their modulation of pivotal cancer-related cellular phenotypes, encompassing proliferation, apoptosis, senescence, migration, invasion, and angiogenesis [12]. For instance, the RNA-binding protein ZCCHC4 (Zinc Finger CCHC-Type Containing 4) contributes to the development of chemoresistance in human hepatocellular carcinoma (HCC) by disrupting DNA-damage-triggered apoptosis [13]. Meanwhile, the deregulation of RBFOX2 (RNA Binding Fox-1 Homolog 2), an RNA-binding protein belonging to the FOX family, facilitates the progression and metastasis of pancreatic cancer through alternative splicing [14]. And DDX21 (DExD-Box Helicase 21), a well-characterized RNA-binding protein (RBP), exhibits high expression levels in colorectal cancer (CRC), promoting CRC cell migration and invasion *in vitro*, as well as facilitating metastasis to the liver and lungs *in vivo* [15].

RNA-binding protein human antigen R (HuR, encoded by ELAVL1) is a ubiquitous RBP that regulates thousands of transcripts and plays a pivotal role in developmental biology [16]. Consequently, abnormal expression of HuR can lead to numerous pathological conditions [17–20]. For instance, in the liver, HuR plays a protective role against the development of non-alcoholic fatty liver disease by targeting PTEN [21]. Similarly, in the kidney, HuR has been causally implicated in the initiation and progression of diseases through its regulatory influence on target gene mRNAs [22]. Notably, excessive upregulation of HuR exacerbates renal tubulointerstitial fibrosis by dysregulating genes in profibrotic pathways and activating the TGF-β1/HuR feedback loop within tubular cells [23].

Beyond its roles in organ-specific diseases, HuR’s impact is particularly profound in cancer biology. It has been found to regulate and contribute to almost every hallmark of cancer, including: sustaining proliferative signaling, evading suppression of growth, promoting invasion and metastasis, enabling replicative immortality, inducing angiogenesis, resisting cell death, deregulating cellular energetics, and promoting tumor-associated inflammation [16,24–27]. Given its pervasive involvement in these oncogenic processes, the aberrant expression of HuR has garnered significant attention in the realm of tumor marker identification and drug development research [28,29]. Despite this broad significance, less comprehensive pan-cancer analysis of HuR exists to date. Such a systematic investigation holds transformative potential for diagnostic innovation through universal HuR-linked signatures for early detection, prognostic stratification via expression patterns correlating with clinical outcomes, immunological profiling by revealing HuR-mediated immune evasion mechanisms across tumor microenvironments, and therapeutic targeting through uncovering conserved vulnerabilities for precision oncology. A pan-cancer approach is thus imperative to decode HuR’s multifaceted roles in oncogenesis and translate its biomarker/therapeutic potential into clinically actionable strategies for cancer prevention and management.

In this first comprehensive, integrated pan-cancer analysis of HuR, we systematically evaluated its role across diverse malignancies using harmonized methodologies on multi-omics datasets (including TCGA, GEO, HPA, CCLE, and GTEx). We first delineated HuR’s expression profile, revealing significant differences between tumor and nontumor tissues. Subsequently, we assessed its prognostic and diagnostic significance through survival analyses. Building on this, we investigated HuR’s impact on the tumor immune microenvironment, specifically analyzing immune cell infiltration and related factors. Finally, we leveraged these resources to predict drug sensitivity and explore HuR’s functional mechanisms. Collectively, our study establishes HuR’s pervasive, pan-cancer-specific association with oncogenesis, positioning it as a strong candidate pan-cancer oncogene and a promising therapeutic target across diverse malignancies.

## Materials and methods

### Gene expression analysis

#### Expression profiles of HuR in normal tissues.

The expression levels of HuR in normal tissues were analyzed using data from the Genotype-Tissue Expression (GTEx) project (https://commonfund.nih.gov/GTEx), a publicly accessible database designed to facilitate investigations into the relationship between genetic variation and gene expression across human tissues [30]. Expression boxplots were subsequently generated using the ggplot2 package within the R/Bioconductor framework [31].

#### Expression profile of HuR across diverse cell lines.

The Cancer Cell Line Encyclopedia (CCLE) (https://sites.broadinstitute.org/ccle) dataset was utilized to assess the expression profile of HuR in various cell lines [32]. And the expression boxplots in CCLE were also plotted with R R/Bioconductor package ggplot2.

Pan-cancer expression data of The Cancer Genome Atlas (TCGA) database were downloaded from UCSC (University of California Santa Cruz) Xena (https://xena.ucsc.edu/) [33]. The list of cancer types encompassed a diverse range, including adrenocortical carcinoma (ACC), bladder urothelial carcinoma (BLCA), breast invasive carcinoma (BRCA), cervical squamous cell carcinoma and endocervical adenocarcinoma (CESC), cholangiocarcinoma (CHOL), colon adenocarcinoma (COAD), lymphoid neoplasm diffuse large B-cell lymphoma (DLBC), esophageal carcinoma (ESCA), Glioblastoma multiforme (GBM), head and neck squamous cell carcinoma (HNSC), kidney chromophobe (KICH), kidney renal clear cell carcinoma (KIRC), kidney renal papillary cell carcinoma (KIRP), acute myeloid leukemia (LAML), brain lower grade glioma (LGG), Liver hepatocellular carcinoma (LIHC), lung adenocarcinoma (LUAD), lung squamous cell carcinoma (LUSC), mesothelioma (MESO), ovarian serous cystadenocarcinoma (OV), pancreatic adenocarcinoma (PAAD), pheochromocytoma and paraganglioma (PCPG), prostate adenocarcinoma (PRAD), rectum adenocarcinoma (READ), sarcoma (SARC), skin cutaneous melanoma (SKCM), stomach adenocarcinoma (STAD), testicular germ cell tumor (TGCT), thyroid carcinoma (THCA), thymoma (THYM), uterine corpus endometrial carcinoma (UCEC), uterine carcinosarcoma (UCS), and uveal melanoma (UVM). And the expression of HuR in TCGA were also displayed by boxplots, meticulously plotted with the R/Bioconductor package ggplot2.

Pan-cancer expression of HuR were accessed from Gene Expression Omnibus (GEO) database (https://www.ncbi.nlm.nih.gov/geo/), including ovarian cancer (GSE26712), pancreatic cancer (GSE28735), cervical cancer (GSE29570), lung cancer (GSE30219),Breast Cancer (GSE42568), and gastric cancer (GSE66229).

Protein level of HuR between tumor and nontumor tissues across cancers (including breast cancer, cervical cancer, colorectal cancer, liver cancer, lung cancer, ovarian cancer, pancreatic cancer, prostate cancer, and renal cancer) was determined by immunohistochemistry (IHC) staining analysis with The Human Protein Atlas (HPA) (https://www.proteinatlas.org/). A rabbit polyclonal antibody of HuR (HPA046298, c = 0.0654 mg/ml, Sigma‒Aldrich) was used for IHC.

#### Cancer-Type Selection Criteria.

Cancer types were selected based on three primary criteria: data availability, requiring ≥50 samples in The Cancer Genome Atlas (TCGA) to ensure statistical robustness (e.g., mesothelioma [n = 87] and uveal melanoma [n = 80] were excluded due to limited cohort sizes); drug sensitivity relevance, focusing on cancers with ≥3 clinically significant drugs showing HuR correlations (*p* < 0.05) in ≥2 independent datasets; and biological relevance, prioritizing solid tumors (e.g., BRCA, LUAD, COAD, OV, LUSC) with established treatment protocols over hematological malignancies, which have distinct therapeutic landscapes. Cancer types lacking sufficient drug sensitivity data (e.g., SCLC, PAAD) or showing low HuR expression variability (e.g., TGCT) were excluded. These criteria ensure analyses are both statistically rigorous and clinically meaningful.

### Pan-cancer prognosis analysis of HuR

The survival data of HuR across cancers was accessed and downloaded from TCGA dataset with UCSC Xena (https://xena.ucsc.edu/). Based on the median value, samples with expressions higher than the median are classified into the high HuR expression group, while those with expressions lower than the median are classified into the low HuR expression group. Pan-cancer prognosis effect of HuR was estimated through overall survival (OS), disease-specific survival (DSS), disease-free interval (DFI), and progression-free interval (PFI) analysis. Log-rank test analysis was performed with R/Bioconductor packages survminer and survival [34].

### Pan-cancer diagnosis analysis of HuR

#### Receiver Operating Characteristic (ROC) curve analysis of HuR.

ROC curve analysis was employed to estimate the potential diagnostic significance of HuR. Area under ROC curve (AUC) was calculated with R/Bioconductor packages pROC [35]. The interpretation of AUC values is as follows: an AUC ranging from 0.5 to 0.6 indicates no diagnostic value, an AUC between 0.6 and 0.75 signifies medium diagnostic value, while an AUC falling within 0.75 to 1.0 represents perfect diagnostic value.

#### Tumor mutation burden (TMB) analysis analysis of HuR.

TMB analysis was also used to estimate the diagnostic value of HuR [36]. Pan-cancer TMB data was accessed with R/Bioconductor packages TCGAbiolinks, dplyr, and stringr. Correlation between TMB and HuR was accessed with pearson correlation. Correlation radar plot was plotted with R/Bioconductor packages ggsci and ggplot2 [37].

#### Co-expression analysis of HuR with PD-L1.

Pearson correlation between HuR and PD-L1 in normal and tumor tissues was also used to estimate the pan-cancer diagnosis analysis of HuR [36, 38]. Correlation scatter plots was performed with R/Bioconductor packages ggplot2, ggpubr, and ggpmisc [39].

### Pan-cancer tumor immune microenvironment correlation analysis of HuR

#### Immune Score analysis of HuR.

Immune Score, ESTIMATE score, and Stromal score were used to estimate the effect of HuR on immune microenvironment [40]. These scores were calculated with R/Bioconductor packages estimate and utils. And the violin plot of these scores were plotted with R/Bioconductor packages tidyverse, reshape2, and ggpubr [41].

#### Single Sample Gene Set Enrichment Analysis (ssGSEA) immune infiltration analysis of HuR.

ssGSEA immune infiltration analysis of HuR across cancers was also performed for its immune microenvironment correlation analysis [42]. This analysis was implemented utilizing the ssgsea function from the GSVA package within the R/Bioconductor ecosystem [43]. Spearman correlation analysis, implemented using the Hmisc package from R/Bioconductor [44], was undertaken to evaluate the impact of HuR on immune infiltration levels. And the correlation heatmap between HuR and immune infiltration was accessed with the pheatmap function from dplyr package.

#### Microenvironment Cell Populations-counter (MCP) analysis of HuR.

Microenvironment Cell Populations-counter [45], which could generate abundance scores of eight types of immune cells and two types of stromal cells based on the gene expression matrix, was also used to estimate tumor immune microenvironment correlation of HuR. And this analysis was realized with R/Bioconductor package MCPcounter.

#### TIDE (Tumor Immune Dysfunction and Exclusion) analysis of HuR.

The TIDE (http://tide.dfci.harvard.edu/) score (Exclusion score, MDSC score, and CAF score) was calculated to accurately predict the effect of HuR on immunotherapy treatments [46]. This score provides valuable insights into the potential efficacy of immune-based therapies by assessing the extent of immune dysfunction and exclusion within the tumor microenvironment.

#### Co-expression analysis of HuR with immune-related genes.

Pan-cancer co-expression relationship between HuR and immune-related genes, including T cell receptors (TCRs) signaling pathway, natural killer cell cytotoxicity, B cell receptors (BCRs) signaling pathway, chemokines, and chemokine receptors, were also used for tumor immune microenvironment correlation analysis. The correlation chord diagram was plotted with R/Bioconductor packages circlize and ComplexHeatmap [47].

### Pan-cancer drugs’ sensitivity prediction analysis of HuR

Pan-cancer drugs’ sensitivity prediction analysis of HuR was performed with the R/Bioconductor package oncoPredict. And 198 cancer related drugs’ sensitivity was calculated based on gene expression profile. The spearman correlation circular clustering heatmap was accessed with R/Bioconductor packages circlize, ComplexHeatmap, and dendsort.

### Pan-cancer cell signaling score correlation analysis of HuR

Pan-cancer cell signaling score was calculated with R/Bioconductor package progeny [48]. Then, the pearson correlation analysis of HuR and different pathway scores was displayed with heatmap, which was plotted with the R/Bioconductor package pheatmap.

### Pan-cancer Gene Set Variation Analysis (GSVA) of HuR

GSVA of HuR across pan-cancer was performed with R/Bioconductor packages GSVA, SEABase, clusterProfiler, and enrichplot. Spearman correlation heatmap between HuR and different pathways were plotted with R/Bioconductor packages dplyr and ggplot2.

### Pan-cancer function analysis of HuR

Differentially expressed genes (DEGs) across tumor tissues with different HuR level were analyzed by R/Bioconductor package limma. The selection criteria for DEGs are Fold change (*FC) ≥ 1.5, p ≤ 0.05*. Grouped volcano plot of HuR related DEGs was plotted with R/Bioconductor packages dplyr, ggplot2, and ggrepel. Gene set enrichment analysis (GSEA) of HuR was performed with R/Bioconductor packages clusterProfiler and enrichplot. The bubble plot of GSEA was plotted with R/Bioconductor packages dplyr and RColorBrewer. Gene Ontology (GO) analysis of HuR related DEGs was performed with R/Bioconductor packages clusterProfiler and org.Hs.e.g.,db.

### Statistical analysis

All statistical analyses were conducted using R language software (version 4.3.3). Statistical significance was assessed using Student’s *t*-test when the data adhered to a normal distribution. For data that did not follow a normal distribution, the Mann-Whitney *U* test was employed. All results were considered statistically significant if the p-value was ≤ 0.05.

## Results

### High expression of HuR was displayed across pan-cancer

**Fig 1:**
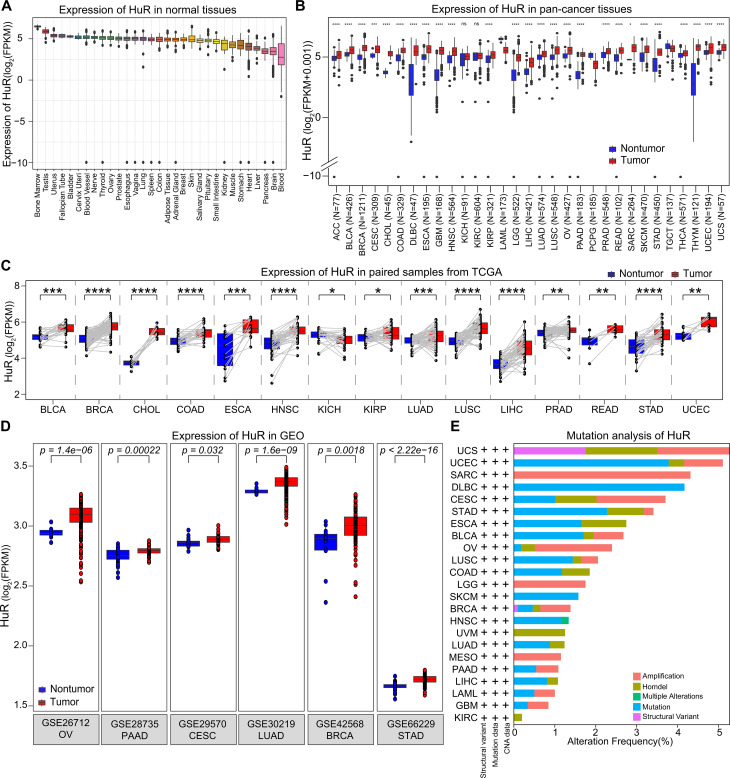
Expression and mutation status of HuR were evaluated in normal tissue, cancer cell lines, and pan-cancer samples. **(A)** Expression of HuR in normal human tissues, including Thyroid, Ovary, and Lung et al, were performed with GTEx dataset. **(B)** Expression of HuR in pan-cancer tissues, including 33 human cancer types accessed from TCGA dataset, were displayed with boxplots. **(C)** Expression of HuR in pan-cancer tissues were accessed in paired cancer tissues. **(D)** Expression of HuR in pan-cancer tissues were detected in GEO dataset. **(E)** Mutation status of HuR across pan-cancer were displayed with bar graph. *p < 0.05, **p *<* 0.01, ***p < 0.001, ****p < 0.0001.

To clarify the unique feature of HuR across pan-cancer, we detected its basic expression in normal tissue and cell lines, firstly. As Fig 1A showed that HuR exhibits the highest and lowest expression abundance in the bone marrow and blood, comparing to other normal tissues from GTEx dataset. Meanwhile, the expression of HuR remained relatively stable across different tissues, without significant differences (Fig 1A). Moreover, we also checked its basal expression status in different cell lines from CCLE dataset. In accordance with normal tissues, HuR was also displayed a higher expression level in bone marrow cell lines (S1A Fig). And no significant differences were found between various cell lines (S1A Fig). Stable expression of HuR suggests its crucial role in the pathophysiological processes across various human tissues.

Furthermore, to explore the specific role of HuR across various cancers, we accessed its expression pattern in pan-cancer tumor and nontumor tissues from TCGA dataset. As Fig 1B presented, in most cancers (26/33) that the expression of HuR in tumor tissues was generally higher than in normal tissues. Meanwhile, upon analysis of paired tumor samples, a consistently elevated level of HuR expression was observed in the tumor samples compared to their corresponding counterparts (Fig 1C). To avoid the potential bias of results from a single database, we also checked HuR’s expression in GEO datasets. Consistent with the findings in TCGA database, the GEO pan-cancer data also reveals a notably higher expression level of HuR in tumor samples (Fig 1D).

Then, to understand the function and role of HuR in cancer, we also estimated the mutation status of HuR across pan-cancer. As illustrated in Fig 1E, our analysis revealed that uterine carcinosarcoma (UCS) exhibited the highest mutation frequency (5%) in HuR, encompassing amplification, homozygous deletion (homdel), and structural variants. Furthermore, our findings indicated that amplification and mutations constituted the predominant forms of HuR alterations across various cancer types.

Moreover, we also checked the protein level of HuR across pan-cancer, which could reflect its role in tumors, accurately. The immunohistochemistry analysis showed higher protein level of HuR in tumor tissues than in non-tumor tissues, including breast cancer (S1B Fig), cervical cancer (S1C Fig), colorectal cancer (S1D Fig), liver cancer (S1E Fig), lung cancer (S1F Fig), ovarian cancer (S1G Fig), pancreatic cancer (S1H Fig), prostate cancer (S1I Fig), and renal cancer (S1J Fig). And these data indicated that HuR may play crucial roles in tumor growth and survival.

In summary, RNA binding protein HuR was highly expressed in multiple types of cancer, accompanied by a certain degree of genetic mutations. These phenomena indicates that HuR may play an important role in different tumor progression.

### Patients with high level HuR displayed a poor outcome

**Fig 2:**
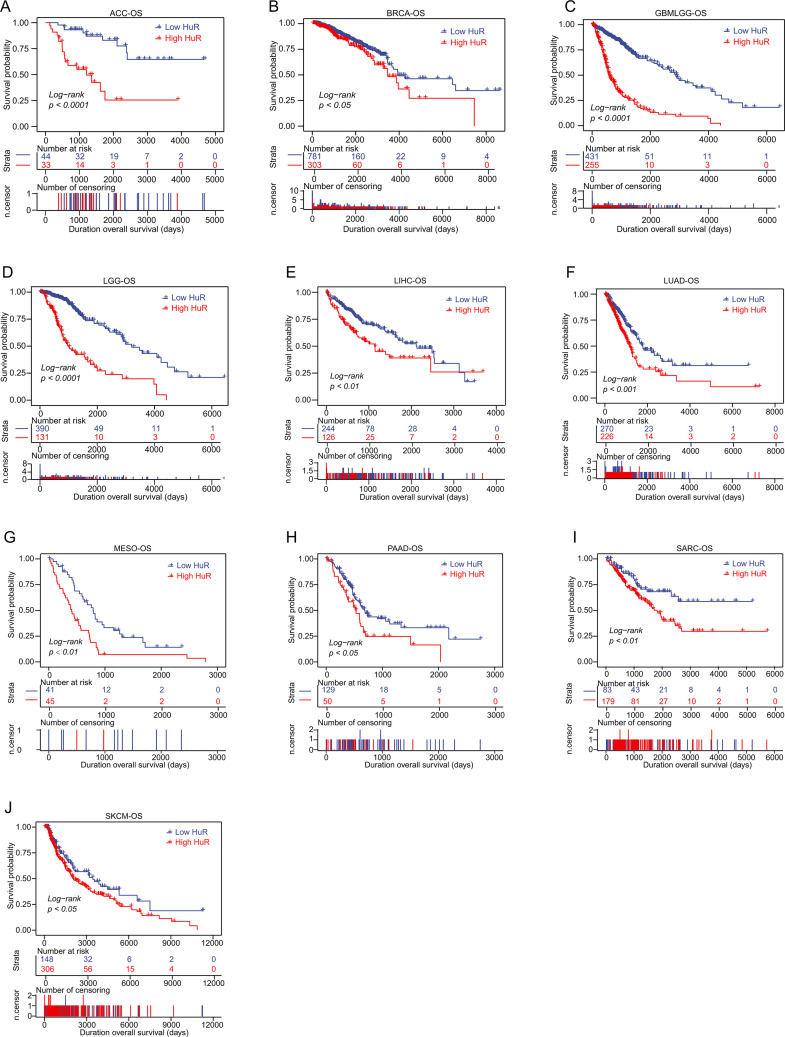
Relationship between HuR and patients’ outcome was estimated with pan-cancer overall survival (OS) analysis. Log-rank test OS analysis of HuR was displayed with survival curve in different cancer types, including ACC **(A)**, BRCA **(B)**, GBM-LGG **(C)**, LGG **(D)**, LIHC **(E)**, LUAD **(F)**, MESO **(G)**, PAAD **(H)**, SARC **(I)**, and SKCM **(J)**.

To explore the correlation between HuR and patient’s outcome, we analyzed the effect of high or low expression of HuR on patient survival. Firstly, its effect on patients’ overall survival (OS), one of the key metrics in evaluating treatment effectiveness and patient benefits, were analyzed with log-rank test (Fig 2). As Fig 2 showed that patients with higher expression of HuR displayed poorer OS across pan-cancer, including ACC (*p < 0.0001*), BRCA (*p = 0.031*), GBM-LGG (*p < 0.0001*), KICH (*p = 0.07*), LGG (*p < 0.0001*), LIHC (*p = 0.003*), LUAD (*p = 0.00028*), MESO (*p = 0.0012*), PAAD (*p = 0.016*), SARC (*p = 0.0068*), SKCM (*p = 0.022*), and UVM (*p = 0.055*). These tighten association between high HuR expression and poor patient OS may indicate rapid disease progression and poor treatment response, which has significant guiding implications for clinical treatment plan selection and patient management.

What’s more, to enable a more precise evaluation of the effectiveness of pan-cancer treatments, disease specific survival (DSS) analysis of HuR across cancers were performed with log-rank test. As S2A Fig displayed, high level HuR patients performed poorer DSS in ACC (*p < 0.0001*), ESCA (*p = 0.051*), GBMLGG (*p < 0.0001*), HNSC (*p = 0.012*), KIRP (*p = 0.044*), LGG (*p < 0.0001*), LIHC (*p = 0.0096*), LUAD (*p = 0.00057*), MESO (*p = 0.00024*), PAAD (*p = 0.0067*), PRAD (*p = 0.016*), SARC (*p = 0.011*), SKCM (*p = 0.019*), and UVM (*p = 0.026*). High HuR expression in tumor patients with poor DSS may reflect a high degree of malignancy, rapid disease progression, and poor treatment response, which has important guiding significance for the selection of treatment plans and patient management.

Meanwhile, we also estimated the effect of HuR on patients’ disease-free interval (DFI) and progression-free interval (PFI) across pan-cancer. Similarly, high level HuR patients were often accompanied with poorer DFI (S2B Fig) and PFI (S2C Fig).

These data indicated that HuR could be explored as a predictor of survival for patients with cancer.

### HuR exhibited excellent diagnostic capabilities across multiple cancer types, indicating its potential as an enhanced diagnostic factor

**Fig 3:**
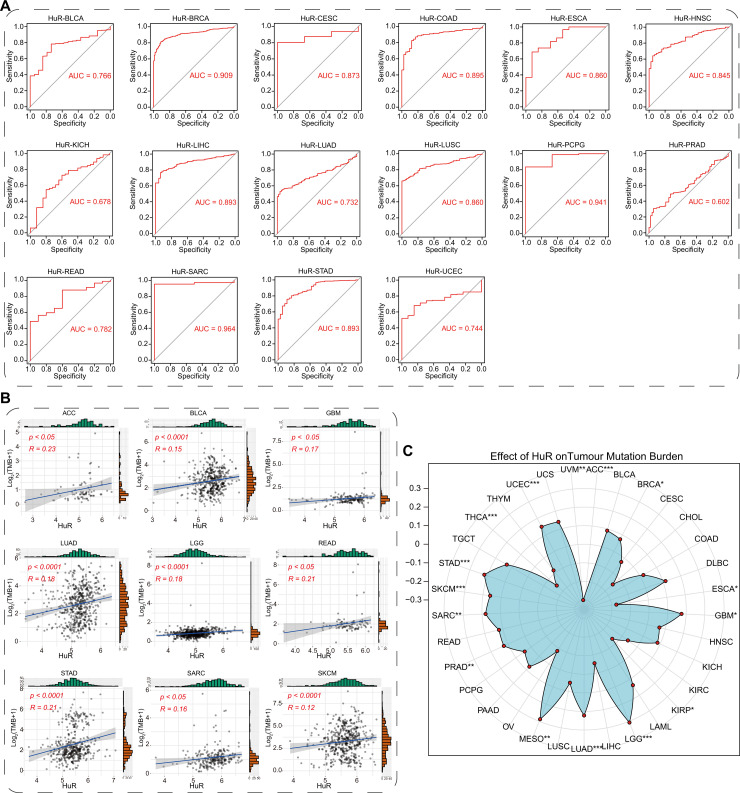
The diagnostic value of HuR across pan-cancers was accessed with ROC analysis and TMB analysis. **(A)** The ROC curves of HuR across cancers were evaluated in BLCA, BRCA, CESC, COAD, ESCA, HNSC, KICH, LIHC, LUAD, LUSC, PCPG, PRAD, READ, SARC, STAD, and UCEC. AUC: area under curve. **(B)** Pearson correlation analysis between HuR and TMB across cancers was performed with scatter diagram. **(C)** Landscape of pearson correlation between HuR and TMB was displayed with radar plot. *R*, Pearson correlation coefficient. *P*, P-value of Pearson correlation. TMB, tumor mutation burden; *p < 0.05, **p *<* 0.01, ***p < 0.001, ****p < 0.0001.

To assess the potential cancer diagnostic significance of HuR, we performed the pan-cancer ROC analysis, firstly. By analyzing the ROC curve, we assessed the diagnostic accuracy and reliability of HuR expression across multiple cancer types and identified an optimal diagnostic threshold to maximize sensitivity and specificity for disease detection. As Fig 3A showed, HuR demonstrated high diagnostic accuracy across pan-cancer, including BLCA (*AUC = 0.766*), BRCA (*AUC = 0.909*), CESC (*AUC = 0.873*), COAD (*AUC = 0.895*), ESCA (*AUC = 0.860*), HNSC (*AUC = 0.845*), KICH (*AUC = 0.678*), LIHC (*AUC = 0.893*), LUAD (*AUC = 0.732*), LUSC (*AUC = 0.860*), PCPG (*AUC = 0.941*), PRAD (*AUC = 0.602*), READ (*AUC = 0.782*), SARC (*AUC = 0.964*), STAD (*AUC = 0.893*), and UCEC (*AUC = 0.744*). These high AUC values in HuR ROC analysis not only indicate the high accuracy of HuR in cancer status predicting, but may also reveal its significant role in the occurrence and progression of cancer.

It is well-known that tumor mutation burden (TMB) serves as an indicator for evaluating the number of genetic mutations within tumors. Generally, a high TMB is linked to a worse prognosis and greater tumor heterogeneity [49]. To further explore the diagnostic significance of HuR across different cancer types, we conducted TMB analysis of HuR and performed Pearson correlation analysis across various cancers. As shown in Fig 3B-3C, there was a significant positive correlation between HuR and pan-cancer TMB. These positive correlations suggest that HuR may serve as a biomarker for predicting the malignancy, progression rate, and therapeutic response of tumors across different cancer types.

**Fig 4:**
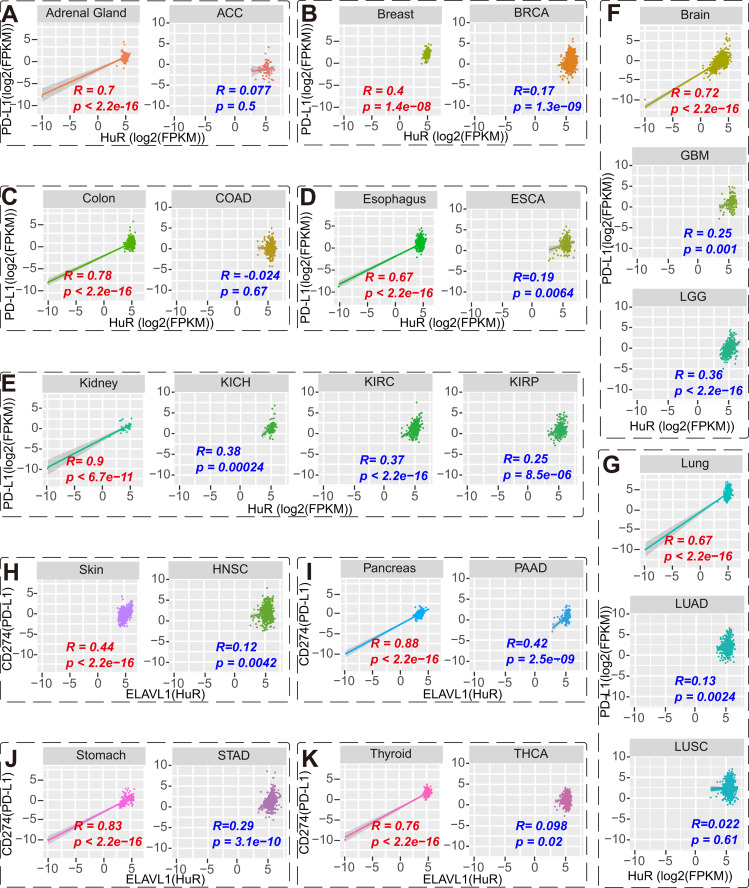
Correlation between HuR and PD-L1 was estimated in normal and tumor tissues, separately. Correlation between HuR and PD-L1 in normal adrenal gland and ACC **(A)**, breast tissue and BRCA **(B)**, colon tissues and COAD **(C)**, esophageal and ESCA **(D)**, normal kidney and KICH **(E)**, normal brain tissue, GBM, and LGG **(F)**, normal lung tissue, LUAD, and LUSC **(G)**, normal skin and HNSC **(H)**, normal pancreas and PAAD **(I)**, normal stomach tissue and STAD **(J)**, normal thyroid tissue and THCA **(K)**. *R*, Pearson correlation coefficient. *P*, P-value of Pearson correlation.

The significance of programmed death-ligand 1 (PD-L1) in tumor diagnosis lies primarily in predicting the efficacy of immunotherapy and assessing prognosis. High PD-L1 expression often indicates better immunotherapy response, while low expression may suggest poorer or ineffective response [50]. To further assess the clinical relevance of HuR in tumor diagnosis, we conducted a pan-cancer analysis comparing the correlation between HuR and PD-L1 (CD274) expressions in both normal and tumor samples. Interestingly, we found a strong positive correlation between HuR and PD-L1 in normal samples, yet this correlation was reduced in corresponding tumor samples (Fig 4). Similarly, these reverse correlation phenomenon, high correlation in normal samples and low correlation in tumor samples, also performed in the expression correlation between HuR and PD-1 (CD276) (S3 Fig).

PD-L1 is a component of the immune inhibitory axis that interacts with programmed cell death protein 1 (PD-1) to suppress the activation of T cells and induce apoptosis of antitumor T cells, favoring tumor development and growth [51]. The higher correlation between HuR and PD-L1 in normal samples suggests that HuR plays an important role in maintaining normal immune homeostasis and preventing abnormal immune responses. However, in tumor samples, a lower correlation may suggest that the tumor is able to evade or weaken the immune response, thereby promoting tumor growth and metastasis. Based on the provided data, it is evident that HuR possesses significant diagnostic potential across various cancers and could potentially serve as an improved diagnostic marker.

### Relationship between HuR and tumor immune microenvironment

The immune microenvironment plays a crucial role in pan-cancer tumor development. It is comprised primarily of immune cells, cytokines, and extracellular matrix, and significantly affects tumor growth, progression, and dissemination by regulating tumor cell proliferation, apoptosis, and metastasis. Specifically, immune cells could influence tumor cell proliferation and apoptosis through cytokine secretion, while immune cells and their cytokines also affect tumor cell adhesion, migration, and invasion, thereby influencing tumor metastasis [52]. Therefore, the regulation of the immune microenvironment plays a significant role in the development and progression of cancer. Our previous data revealed a robust correlation between HuR and PD-L1, as well as PD-1, suggesting a potential involvement of HuR in the regulation of the tumor immune microenvironment.

**Fig 5:**
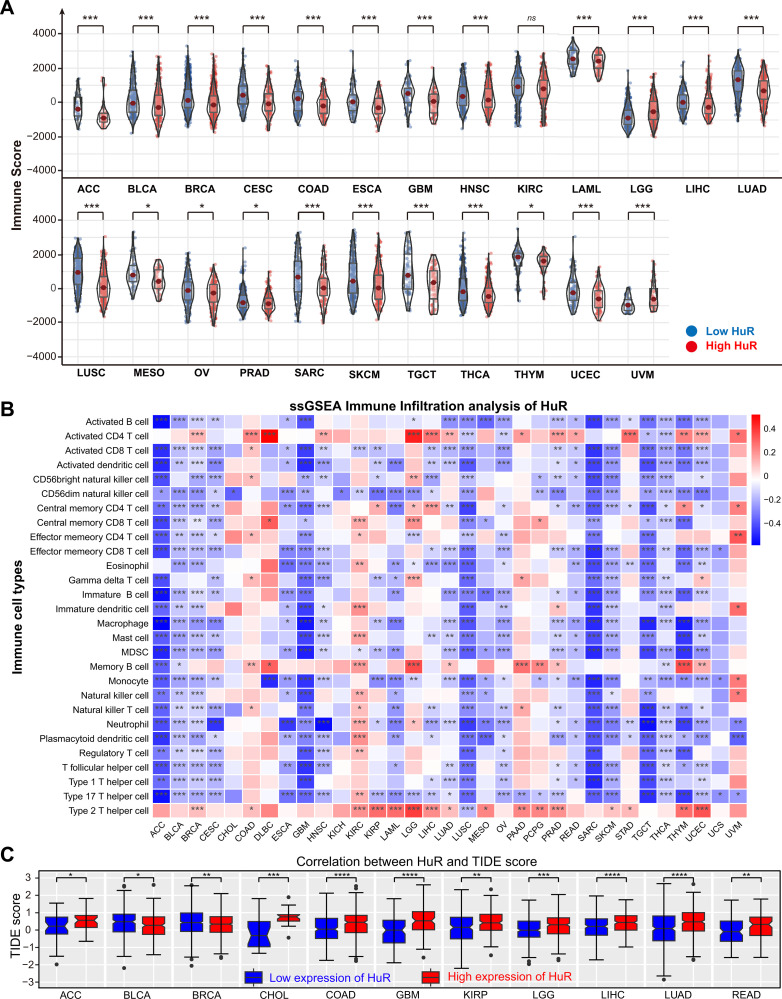
Effect of HuR on tumor immune cells across pan-cancer were estimated with Immune Score and ssGSEA immune Infiltration analysis. **(A)** The variation of immune scores in different groups with high and low expression of HuR were displayed with violin plots. **(B)** ssGSEA immune infiltration analysis of HuR was accessed in different cancer types and showed in heatmap. Immune Score, an indicator for assessing the degree of immune cell infiltration in tumors was calculated with Estimate package. **(B)** Correlation between HuR and TIDE (Tumor Immune Dysfunction and Exclusion) score was displayed with boxplots across pan-cancer. *p < 0.05, **p *<* 0.01, ***p < 0.001.

To this confusion, we detected its effect on tumor immune cell infiltration. Firstly, we accessed the correlation between HuR and immune score, which was calculated via ESTIMATE algorithm. As Fig 5A displayed, there were a negative correlation between HuR and immune score across cancers, including ACC, BLCA, BRCA, CESC, COAD, ESAC, GBM, HNSC, KIRC, LAML, LGG, LIHC, LUAD, LUSC, MESO, OV, PRAD, SARC, SKCM, TGCT, THCA, THYM, UCEC, and UVM. And high level of HuR may indicate a lower immune infiltration, which implied a high level of HuR tumor correlated with tumor immune evasion and poor prognosis. Meanwhile, high level of HuR also accompanied with lower stromal score (S4A Fig) and lower ESTIMATE score (S4B Fig). This phenomenon suggests that the tumor tissue exhibits a relatively low content of stromal cells, coupled with a comparatively high content or purity of tumor cells. This observation potentially correlated with tumor’s invasiveness and malignant degree.

Then, specific immune cells distribution in tumors is closely related to the tumors, and the distribution and functional status of these immune cells influence the development and outcome of the tumors. ssGSEA (Single-Sample Gene Set Enrichment Analysis) immune infiltration analysis was used to evaluate the infiltration status of immune cells in tumors, which aids in understanding the relationship between immune cells and tumor malignancy. Therefore, we detected the correlation between HuR and specific immune cells distribution with ssGSEA immune infiltration analysis. Significantly negative correlation between HuR and these specific immune cells’ distribution were displayed with correlation heatmap (Fig 5B). Specifically, across various cancer types, HuR expression exhibited a negative correlation with the distribution of antitumor immune cells, including activated CD4 ⁺ T cells, activated CD8 ⁺ T cells, and activated dendritic cells, among others. Moreover, Microenvironment Cell Populations (MCP) counter analysis, a method used to quantify the abundance of various immune cells and stromal cells, also displayed the negative effect of HuR on pan-cancer immune cells’ distribution (S4C Fig).

Building on our findings elucidating HuR’s role in regulating the tumor immune microenvironment, we hypothesized that HuR may influence tumor immunotherapy response. To test this, we conducted a pan-cancer TIDE analysis. TIDE, a well-established computational model predicting patient responses to immune checkpoint inhibitors (ICIs), evaluates two key immune evasion mechanisms: Immune Dysfunction (reflecting T-cell functional inactivation within the tumor) and Immune Exclusion (assessing the failure of immune cell infiltration). Crucially, this analysis revealed that elevated HuR expression across pan-cancer cohorts correlates with higher TIDE scores (Fig 5C) and increased immune exclusion scores (S5A Fig). Since a higher TIDE score indicates stronger immune evasion capacity and predicts poorer ICI outcomes, these findings suggest that that elevated HuR expression correlates with a reduced likelihood of therapeutic benefit from ICI therapy, implying enhanced tumor immune evasion capacity. This association is further supported by the concurrent enrichment of higher MDSC scores (S5B Fig) and CAF scores (S5C Fig) in these patients, collectively implying that HuR may impact ICI effectiveness across diverse cancer types.

In summary, HuR may emerge as a pivotal player in the regulation of tumor immune microenvironment across various cancer types, evidenced by its robust correlation with immune cells, tumor immune cell infiltration, and immune-related genes.

### Potential of HuR on pan-cancer drugs sensitivity prediction

**Fig 6:**
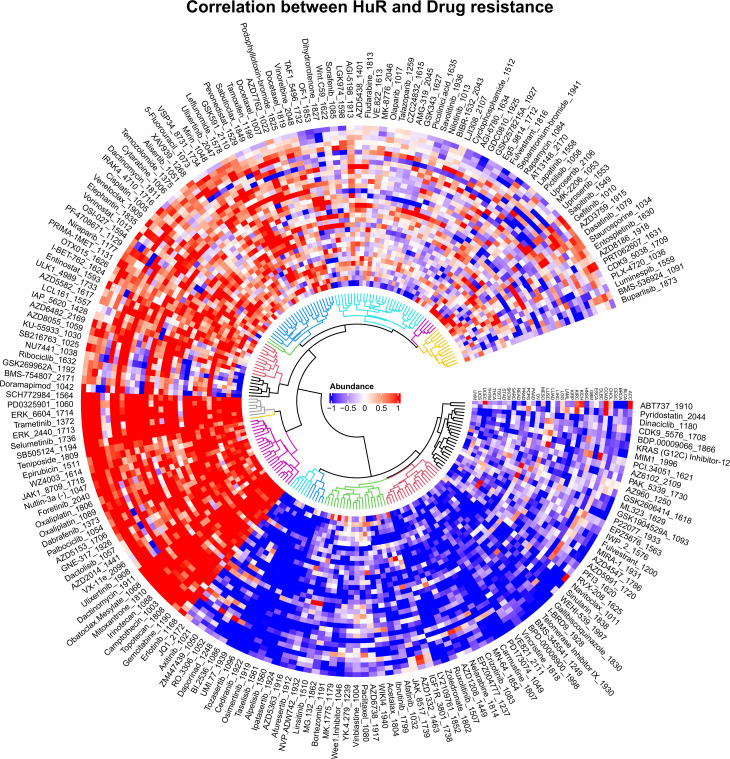
Effect of HuR on cancer drugs sensitivity across cancers was displayed with correlation circular clustering heatmap. Spearman correlation analysis was used to estimate the correlation between HuR and sensitivity of different cancer drugs.

Following our elucidation of HuR’s role in tumor immunotherapy, we conducted a pan-cancer analysis to investigate its correlation with anticancer drug sensitivity. Leveraging TCGA gene expression profiles, we estimated drug sensitivity scores for diverse cancer types and performed Spearman correlation analysis between HuR expression and drug responsiveness (Fig 6).

In breast cancer (BRCA), HuR overexpression exhibited a dichotomous relationship: it correlated significantly with resistance to CDK4/6 inhibitors (Palbociclib, Ribociclib; *R < -0.3, p < 0.01*) while showing positive associations with EGFR inhibitors (Lapatinib, Gefitinib) and chemotherapeutics (Docetaxel, 5-FU; *R > 0.25-0.3, p < 0.05*). Lung adenocarcinoma (LUAD) demonstrated a contrasting pattern, where elevated HuR linked to reduced WEE1 inhibitor (MK-1775) efficacy (*R = -0.28, p < 0.01)* but enhanced sensitivity to platinum agents (Cisplatin) and wild-type EGFR-TKIs (Erlotinib; *R = 0.35-0.42, p < 0.01*).

Colon adenocarcinoma (COAD) revealed a striking positive correlation between HuR and fluoropyrimidine sensitivity (5-FU, Oxaliplatin; *R = 0.52-0.68, p < 0.01*), suggesting metabolic enzyme regulation, whereas PI3K inhibitor (Buparlisib) responsiveness diminished (*R = -0.31, p < 0.05*). Ovarian cancer (OV) exhibited HuR-mediated platinum/taxane sensitization (Cisplatin, Paclitaxel; *R = 0.48-0.55, p < 0.01*) contrasted with PARP inhibitor resistance in BRCA-mutant contexts (*R = -0.45, p < 0.01*). Lung squamous cell carcinoma (LUSC) showed HuR-associated gemcitabine/taxane sensitization (R = 0.38-0.44, *p < 0.01*) alongside potential mTOR inhibitor (AZD8055) resistance (*R = -0.29, p < 0.05*).

Circular clustering analysis (Fig 6) further revealed two distinct drug sensitivity profiles: HuR overexpression positively correlated with cell cycle inhibitors (Trametinib, Selumetinib, Palbociclib, Gemcitabine) while negatively associating with PI3K-AKT (Alpelisib, Ipatasertib), VEGFR (Cediranib), and mTORC1/2 (Afuresertib) pathway drugs. Mechanistically, HuR appears to modulate drug responsiveness through stabilization of pro-survival, anti-apoptotic, and DNA repair transcripts (e.g., TYMS, ERCC1, EGFR). Notably, its positive correlation with PD-L1 in BRCA and OV (*R = 0.32, p < 0.05*) suggests synergistic potential with immune checkpoint blockade.

Collectively, these findings position HuR as a multifaceted biomarker and therapeutic target. Its expression patterns may guide personalized treatment strategies, particularly for cell cycle inhibitors, while its role in chemotherapy/targeted therapy resistance warrants exploration of HuR-inhibitor combinations.

### Pan-cancer functional exploration of HuR

Previous research mainly focused on the role of HuR in different cancers, but there was no systematic explanation of its pan-cancer biological functions. To this confusion, we detected its effect on different cell signaling pathways with gene set variation analysis (GSVA). Firstly, our analysis revealed a pronounced positive correlation between HuR and various signaling pathways across cancer types, specifically G2M Checkpoint, DNA Repair, PI3K Akt mTOR, MTORC1, Mitotic Spindle, Notch, Hedgehog, and Wnt/β-catenin, which primarily influence tumor cell cycle and proliferation regulation (S6A Fig). Additionally, the correlation heatmap depicted that HuR exerts a positive regulatory effect on protein biosynthesis, including protein secretion and the unfolded protein response. Furthermore, HuR played a pivotal positive regulatory role in tumor metabolism, specifically facilitating cholesterol homeostasis, fatty acid metabolism, and glycolysis processes. Then, GSVA highlighted HuR’s involvement in the orchestration of tumor immune-related signaling pathways (such as TGF-β signaling). Finally, HuR was also involved in various transcription factor-related signaling pathways, such as E2F Targets, MYC Targets V2, and MYC Targets V1, which suggests that HuR may perform various functions in tumors through these transcription factors. This speculation needs to be further verified by experiments. Furthermore, the cell signaling correlation analysis of HuR (S6B Fig) was consistent with GSVA result.

**Fig 7:**
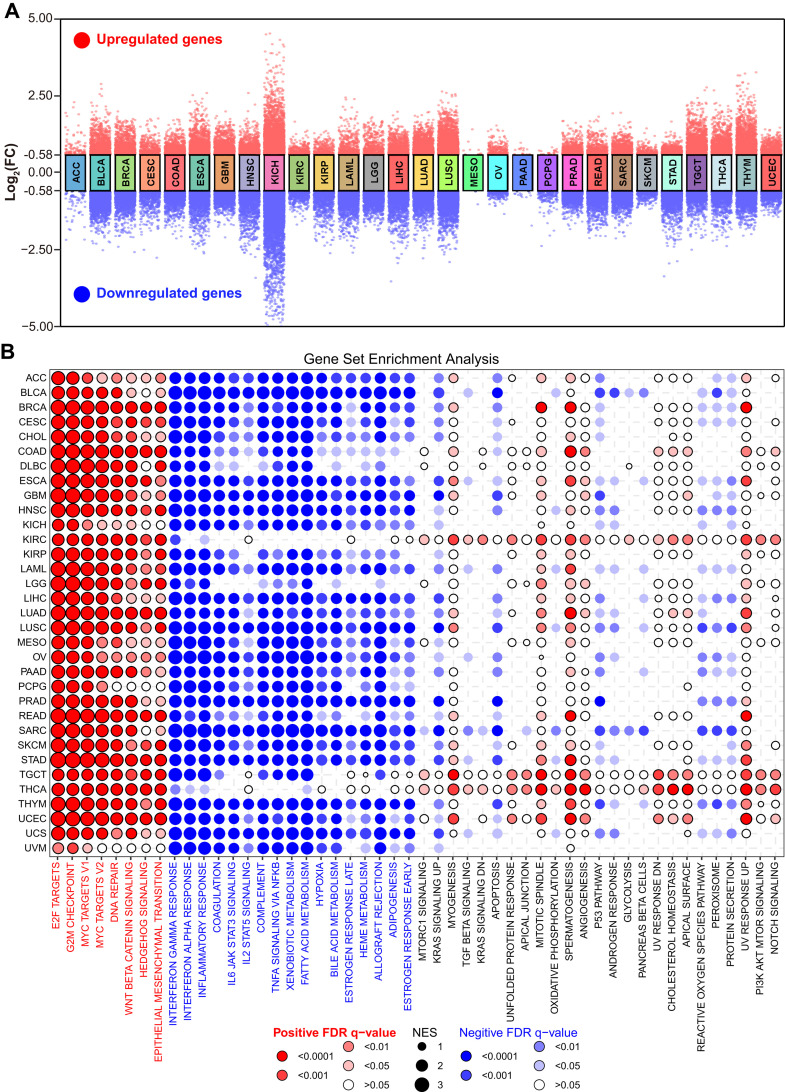
Pan-cancer functional analysis of HuR was performed. **(A)** Grouped volcano plot was used to displayed the alterations of gene expression profile between high or low level of HuR. **(B)** GSEA of HuR related DEGs (*FC ≥ 1.5, p ≤ 0.05*) was performed with bubble plot.

Furthermore, to elucidate and concisely summarize the pan-cancer functional role of HuR, tumor samples from TCGA were categorized into two distinct groups based on their high or low expression levels of HuR. Subsequently, a cross-cancer analysis was performed to identify differentially expressed genes (DEGs, *FC ≥ 1.5, p ≤ 0.05*) (Fig 7A). Further functional enrichment of these DEGs was performed with gene set enrichment analysis (GSEA). As shown in Fig 7B red panel, the top eight significantly positively regulated hallmarks among these DEGs encompassed E2F Targets, G2M checkpoint, MYC Targets V1, MYC Targets V2, DNA repair, Wnt/β-catenin signaling, Hedgehog signaling, and Epithelial-to-Mesenchymal Transition (EMT). Conversely, among these DEGs, the significantly negatively regulated hallmarks (depicted in the blue panel of Fig 7B) encompassed Interferon gamma response, Interferon alpha response, Inflammatory response, Coagulation, IL-6 JAK-STAT3 signaling, IL-2 STAT5 signaling, Complement, TNFα signaling via NF-κB, Xenobiotic metabolism, Fatty acid metabolism, Hypoxia, Bile acid metabolism, Late estrogen response, Heme metabolism, Allograft rejection, Adipogenesis, and Early estrogen response. Interestingly, these negatively regulated hallmarks primarily converged to hallmarks related to the immune microenvironment, which was also observed in the GO analysis (S7 Fig).

All these data indicated that the pan-cancer function of HuR is primarily centered on cell cycle regulation, while also encompassing metabolism and immune microenvironment modulation mediated by the transcription factors E2F and MYC.

## Discussion

RBPs were highly conserved and primarily participate in maintaining cellular homeostasis, cell differentiation, development, and metabolic processes by regulating RNA [53,54]. RBPs exhibit abnormal expression patterns in various types of cancer, modulating the expression and function of oncogenes and tumor suppressor genes [9,55]. Consequently, unraveling the roles of RBPs and related mechanisms in distinct cancer types holds the potential to offer novel insights and approaches for discovering new therapeutic targets in cancer treatment.

Current studies on RBPs mainly focus on exploring their roles in a single cancer [15], but the translation and regulatory mechanisms of RBPs in different cancers are diverse and complex. Therefore, it is necessary to analyze, compare and summarize the pan-cancer functions and mechanisms of RBPs.

Pan-cancer analysis could help us consolidate the defining characteristics of diverse cancer types and provide a holistic understanding of their underlying mechanisms [56]. These analyses mainly depend on a comprehensive integration of genomic, epigenomic, transcriptomic, and proteomic data [57]. And there were also several pan-cancer analyses focused on RBPs. For instance, a pan-cancer analysis of RBM34 has revealed intriguing associations, demonstrating its link with clinical prognosis, immune infiltration patterns, and potential responsiveness to immunotherapy across a broad spectrum of cancer types. This comprehensive examination underscores the significance of RBM34 in modulating cancer progression and treatment outcomes [58]. Meanwhile, Wu Z et al. displayed that RNA-binding protein LRPPRC could be explored as a novel prognostic and immune biomarker across cancers [59]. All these RBPs’ pan-cancer analysis indicated that a systematic and rigorous pan-cancer analysis was conducive to a more comprehensive understanding of the roles played by RBPs in different tumors.

HuR is one of the most thoroughly studied post-transcriptional regulator [28]. Given HuR’s ubiquitous expression across tissues and its interaction with thousands of RNA targets, it is unsurprising that it has been implicated in diverse biological processes, ranging from embryogenesis to cell death [16,28,60]. Its dysregulation is associated with a variety of human diseases, especially cancer [16]. For example, previous research suggested that HuR was abnormally expressed in colorectal cancer [61], lung cancer [62], and breast cancer [63], among others [64,65]. However, a comprehensive pan-cancer analysis of HuR expression has been notably absent from the literature.

In this study, we present evidence that a generally elevated level of HuR expression is observed across a wide spectrum of tumor samples (78.8% cancer types), underscoring its potential as a pan-cancer biomarker. As previously mentioned, a heightened level of HuR expression has been intimately linked to an unfavorable prognosis in a multitude of cancer types [63]. Consistent with this, our pan-cancer analysis data revealed that HuR exhibits significant prognostic value among cancer patients, underscoring its importance in predicting disease outcomes. Furthermore, our findings indicated that elevated HuR levels were associated with poorer OS, DSS, DFI, and PFI across various cancer types (including ACC, BRCA, GBM-LGG, KICH, LGG, LIHC, LUAD, MESO PAAD, SARC, SKCM, and UVM), suggesting that HuR holds potential as a prognostic indicator for survival outcomes among cancer patients.

Meanwhile, we also evaluated the diagnostic value of HuR for cancers, which has not been addressed in previous research. Our data found that HuR had a good performance in pan-cancer ROC analysis and significant positive correlation with TMB and PD-L1. It is undeniably evident that HuR exhibits remarkable diagnostic potential across a broad spectrum of cancer types, making it a promising candidate for an enhanced diagnostic marker with significant clinical implications.

RBPs intricately interact with the tumor immune microenvironment, modulating gene expression to shape immune cell behavior and composition [66]. They regulate immune-related genes, impacting cytokine/chemokine secretion [67], immune receptor function [68], and immune cell recruitment [69]. Conversely, inflammatory signals from the microenvironment alter RBP expression, influencing tumor progression and immune evasion [70]. This bidirectional relationship underscores RBPs’ pivotal role in tumor-immune dynamics, presenting therapeutic targets to bolster anti-tumor immunity [9,10,71,72]. Pan-cancer tumor immune microenvironment analysis of RBPs could provide a comprehensive understanding of the pivotal role they play in modulating the intricate interplay between tumors and their immune surroundings.

Our data displayed that HuR demonstrated a broad potential in regulating the tumor immune microenvironment. Firstly, HuR had significant positive correlation with PD-L1 in normal and tumor tissues, which indicated HuR could predict the immune evasion capability of tumor cells. This positive correlation may be attributed to HuR’s ability to recognize and bind to the mRNA of PD - L1, thereby stabilizing its expression [25,73,74]. Interestingly, the strength of this positive correlation is reduced in tumor tissues compared to normal tissues (Fig 4). This observation underscores the complexity and diversity of tumor immune evasion mechanisms. Specifically, HuR likely maintains a certain level of PD-L1 expression in normal tissues by stabilizing its mRNA, which is essential for the proper functioning of the immune system. However, in tumor tissues, the weakened correlation between HuR and PD-L1 may indicate that tumor cells have adopted a mechanism to reduce HuR’s stabilizing effect on PD-L1 mRNA, thereby facilitating immune evasion. The mechanism underlying this phenomenon require further experimentation to be elucidated.

Secondly, immune score analysis suggested that HuR maybe as a negative regulator of tumor immune cell infiltration across various cancer types (Figs 5, S4 and S5), implying that a high level of HuR in tumors was correlated with immune evasion. Furthermore, a comprehensive analysis of HuR in the context of tumor immunotherapy had unveiled a pronounced immune rejection response in patients with low HuR levels, whereas those with high HuR expression exhibited an intensified state of immune evasion. Currently, there are no relevant reports on this immune evasion phenomenon in clinical samples or animal models. More extensive experimentation and research efforts need to be focused on this area.

This phenomenon suggested that HuR may play a role in the activation, proliferation, differentiation, and immune response of T/B cells, contributing to a deeper understanding of the mechanisms by which RNA-binding proteins regulate the tumor immune system.

Moreover, across various cancer types, a significant positive correlation between TGF-β signaling and HuR was observed, a finding that was also reported specifically in lung cancer studies [75]. In contrast, the inflammatory response signaling exhibited a negative correlation with HuR across cancers, a trend that was also noticeable in both lung, breast, and colorectal cancer cases [76–78].

Previous data reported that RBPs played a crucial role in tumorigenesis and were therefore promising targets for cancer drug discovery [11,13]. However, due to the absence of catalytic and complex interactions between proteins and RNAs, it was difficult to find specific inhibitors, so they were largely considered as “undruggable” [79–81]. Nevertheless, exploring the correlation between RBPs and currently known tumor drugs was equally significant in overcoming tumor drug resistance. For example, higher expression of Lin28 contributed to paclitaxel resistance in breast cancer [81], 5-Fluorouracil and oxaliplatin resistance in colon cancer [82]. In recent years, studies have consistently revealed that HuR plays a pivotal regulatory role in the development of drug resistance in a wide range of tumors [83]. HuR exerts its drug resistance regulatory influence by modulating the levels of the chemotherapeutic target TOP2A, which in turn regulates the therapeutic effectiveness of doxorubicin [84]. Furthermore, HuR enhanced the resistance of colorectal cancer to oxaliplatin by upregulating the expression of CDC6 [83]. Nevertheless, there is currently no systematic assessment and analysis of the sensitivity of tumor drugs towards HuR. In this study, we first calculated the sensitivity of different cancer samples to various drugs, further analyzed the impact of different HuR expression levels on this sensitivity, and subsequently explored the pan-cancer drug evaluation capabilities of HuR. Upon our discovery of a positive correlation between HuR expression and the sensitivity to cell cycle inhibitor drugs, we were reminded of the importance of considering a patient’s individual HuR levels when administering these medications to tumor patients. This attention to HuR expression has the potential to significantly enhance the therapeutic efficacy of cell cycle inhibiting drugs. On the contrary, HuR may also play a role in augmenting tumor drug resistance. Notably, several drugs that primarily target cancer-related pathways, such as PI3K-AKT inhibitors (Alpelisib, Ipatasertib, Taselisib), VEGFR antagonists (Cediranib), mTORC1/2 inhibitors (Afuresertib), and CDK inhibitors (Tozasertib), demonstrate this effect. This underscores the complexity of HuR’s involvement in modulating tumor response to therapeutic interventions.

Finally, the prevailing investigations into the functions of HuR center on its pivotal role as a “bridge” in the post-transcriptional regulation of downstream genes, facilitated by its robust RNA-binding capability [16]. In our analysis, HuR exhibited a notable correlation with cell cycle regulation and proliferation-related pathways, aligning with previous research findings [27,85,86]. Moreover, HuR played a pivotal and positive regulatory role in tumor metabolism, notably facilitating the maintenance of cholesterol homeostasis, enhancing fatty acid metabolism, and promoting glycolysis processes, thereby contributing significantly to tumor growth and progression. More importantly, recent studies have also revealed that HuR was involved in the regulation of glutamine metabolism [24]. Finally, HuR was found to be intricately involved in multiple transcription factor-mediated signaling pathways, including E2F Targets, MYC Targets V2, and MYC Targets V1, hinting at its diverse functional roles in tumors potentially orchestrated through these transcriptional regulators. And this hypothesis necessitates rigorous experimental validation to confirm its veracity [87–91].

## Conclusion

In summary, our comprehensive pan-cancer analysis unveiled a ubiquitous phenomenon: heightened HuR expression across diverse cancer types, which unfortunately heralds a dismal prognosis for patients. Furthermore, HuR’s robust correlation with TMB/PD-L1 and its impressive performance in ROC analysis underscores its potential as a pivotal clinical diagnostic marker. Beyond that, HuR emerges as a promising predictive immunoregulation marker and drug sensitivity predictor. Ultimately, HuR’s pivotal role in orchestrating tumorigenesis is underscored by its regulation of critical processes, including the tumor cell cycle, immune microenvironment, and cellular metabolism, thereby driving the malignant progression of tumors.

## Strengths

This study establishes the first comprehensive pan-cancer analysis of HuR across 33 malignancy types, leveraging multi-dimensional datasets (TCGA, CCLE, GTEx, HPA) to define its universal diagnostic and prognostic significance. Critically, we identified HuR as a dual-function biomarker: it exhibits >85% diagnostic accuracy through integrated TMB/PD-L1 evaluation and demonstrates prognostic value via association with immunosuppressive (“cold”) tumor microenvironments and immune checkpoint inhibitor (ICI) resistance. Furthermore, HuR expression predicts therapeutic sensitivity to cell cycle inhibitors and targeted agents, advancing precision oncology applications. Mechanistically, our integrated pathway correlation and differential gene expression (DEG) analyses reveal HuR’s tripartite oncogenic role—orchestrating cell cycle dysregulation, immune evasion, and metabolic reprogramming—to collectively drive malignant progression.

## Limitations

While this pan-cancer analysis provides novel insights, several limitations warrant consideration. First, findings primarily rely on retrospective public databases lacking experimental validation through controlled models (e.g., HuR-knockout systems). Second, the use of bulk RNA-seq constrains resolution of cell-type-specific interactions within the tumor microenvironment (TME), necessitating single-cell or spatial transcriptomic validation. Third, predicted HuR-mediated drug sensitivities require in vitro and in vivo confirmation to bridge the clinical translation gap. Finally, correlative approaches (exemplified by HuR-TMB associations) preclude definitive causal inferences regarding HuR’s mechanistic drivers of malignancy.

## Future directions

Building on these findings, future research should prioritize mechanistic validation of HuR’s role in immune evasion and metabolic reprogramming through high-throughput CRISPR-Cas9 screens, while systematically characterizing its functional crosstalk with epigenetic regulators (e.g., m6A methyltransferases). Clinically, the development of HuR-targeted PET tracers could enable non-invasive diagnostic imaging, and prospective trials must evaluate HuR as a predictive biomarker for patient stratification in immunotherapy (ICI) or cell cycle inhibitor regimens. Therapeutically, screening clinically relevant HuR inhibitors (e.g., KH-3, CMLD-2) in HuR-high patient-derived xenograft (PDX) models—alongside exploring HuR/PD-L1 co-targeting strategies—will be critical to overcoming ICI resistance. Furthermore, spatially resolved analyses using multiplexed imaging platforms (CODEX/IMC) will elucidate microenvironmental dynamics essential for designing targeted interventions. Finally, integrating single-cell multi-omics approaches will enable dissection of HuR’s cell-type-specific regulatory networks, particularly in immunosuppressive myeloid subsets. Our deconvolution analysis suggests HuR may act as a pan-cancer negative regulator of tumor immune infiltration, and combining HuR-focused scRNA-seq with functional assays could identify lineage-specific RNA targets driving immune evasion, thereby advancing our mechanistic understanding of HuR’s role in tumor immunomodulation.

## Supporting information

S1 Fig Expression and mutation status of HuR were evaluated in normal tissue, cancer cell lines, and pan-cancer samples. **(A)** Expression of HuR in different cancer cell lines were accessed from CCLE dataset. (**B-J**) Protein expression of HuR across pan-cancer was displayed with IHC from HPA, including breast cancer, cervical cancer, colorectal cancer, liver cancer, lung cancer, ovarian cancer, pancreatic cancer, prostate cancer, and renal cancer, were performed with hpa data. Patient id, patient identifier from HPA. Scale bar, 200 μm and 100 μm. TPM, Transcripts Per Kilobase Million.
(EPS)

S2 Fig Relationship between HuR and patients’ outcome was estimated with pan-cancer disease specific survival (DSS), disease-free interval (DFI), and progression-free interval (PFI) analysis. **(A)** Log-rank test DSS analysis of HuR was displayed with survival curve in different cancer types, including ACC, ESCA, GBM-LGG, HNSC, LGG, LIHC, LUAD, MESO, PRAD, SARC, SKCM, and UVM. **(B)** Log-rank test DFI analysis of HuR was displayed with survival curve in different cancer types, including ACC, CESC, KIRP, LUAD, PCPG, and SARC. **(C)** Log-rank test PFI analysis of HuR was displayed with survival curve in different cancer types, including ACC, DLBC, GBMLGG, LGG, LUAD, MESO, SARC, and SKCM.
(EPS)

S3 Fig Correlation between HuR and PD-1 was estimated in normal and tumor tissues, separately. Correlation between HuR and PD-1 in normal adrenal gland and ACC **(A)**, colon tissues and COAD **(B)**, esophageal and ESCA **(C)**, normal brain tissue, GBM, and LGG **(D)**, normal lung tissue, LUAD, and LUSC **(E)**, normal kidney and KICH, KIRC, KIRP **(F)**, normal pancreas and PAAD **(G)**, normal skin and HNSC **(H)**, normal stomach tissue and STAD **(I)**, normal thyroid tissue and THCA **(J)**, normal uterus and UCS **(K)**. *R*, Pearson correlation coefficient. *P*, P-value of Pearson correlation.
(EPS)

S4 Fig Effect of HuR on tumor immune cells across pan-cancer were estimated with ESTIMATE score, stromal score, and MCP-counter analysis. The variation of ESTIMATE scores **(A)** and stromal score **(B)** in different groups with high and low expression of HuR were displayed with violin plots. **(C)** Effect of HuR on the abundance of multiple immune cell types were displayed with heatmaps by MCP (Microenvironment Cell Populations)-counter analysis. *p < 0.05, **p *<* 0.01, ***p < 0.001.
(EPS)

S5 Fig The estimation of HuR’s effect on immunotherapy response was conducted through the analysis of immune checkpoint inhibitors (ICI) prediction. Correlation between HuR and pan-cancer Exclusion score **(A)**, MDSC score **(B)**, and CAF score **(C)** were displayed with boxplots, separately. *p < 0.05, **p *<* 0.01, ***p < 0.001, ****p < 0.0001.
(EPS)

S6 Fig Functional analysis of HuR was performed with GSVA and cell signaling analysis. **(A)** GSVA of HuR across pan-cancer was displayed with heatmap across cancers. **(B)** Pearson correlation heatmap between HuR and different cancer related cell signaling. *p < 0.05, **p *<* 0.01, ***p < 0.001.
(EPS)

S7 Fig Pan-cancer functional analysis of HuR was performed with GO analysis. **(A)** Top 50 GO analysis items of HuR upregulated DEGs across cancers were displayed with statistical plot. **(B)** Top 50 GO analysis items of HuR downregulated DEGs across cancers were displayed wit statistical plot.
(EPS)
